# Maturity-Related Differences in Muscle Architecture in Growing Female Volleyball Athletes: A Cross-Sectional Study

**DOI:** 10.3390/children12101415

**Published:** 2025-10-20

**Authors:** Ioli Panidi, Gregory C. Bogdanis, Anastasia Donti, Vasiliki Gaspari, Dimitra Kanna, Gerasimos Terzis, Olyvia Donti

**Affiliations:** School of Physical Education & Sport Science, National and Kapodistrian University of Athens, 17237 Athens, Greece; ipanidi@phed.uoa.gr (I.P.); gbogdanis@phed.uoa.gr (G.C.B.); adonti@phed.uoa.gr (A.D.); vgaspari@phed.uoa.gr (V.G.); dimitrakanna@phed.uoa.gr (D.K.); gterzis@phed.uoa.gr (G.T.)

**Keywords:** architectural characteristics, ultrasound, muscle fascicle, pennation angle, muscle geometry, maturation, volleyball

## Abstract

**Highlights:**

**What are the main findings?**

• Across maturity groups, large differences were observed between pre- and post-PHV, which is a critical time frame for lower limb muscle growth longitudinally and transversely.
• From pre- to circa-PHV, vastus lateralis fascicular length demonstrated greater values than that of gastrocnemius medialis.
• Differences were observed between circa- and post-PHV only in gastrocnemius medialis fascicle length.
• Muscle thickness continued to be progressively larger across subsequent maturity stages.

**What is the implication of the main finding?**

• Skeletal muscle architecture is closely associated with body dimensions that impose mechanical loading and stretching.
• Examining differences in muscle morphology across maturity stages provides insight into neuromuscular performance in developing athletes and supports stage-appropriate training interventions.

**Abstract:**

**Background/Objectives:** This cross-sectional study examined gastrocnemius medialis (GM) and vastus lateralis (VL) muscle architecture in pre-, circa- and post-peak height velocity (PHV) female volleyball athletes. **Methods**: Muscle architecture (fascicle length: FL; pennation angle: PA; muscle thickness: TH) was assessed in 144 athletes using ultrasonography. Stature, body mass, femur and calf length were measured. Maturity index was calculated from anthropometrics. Athletes were classified as pre-PHV, circa-PHV and post-PHV. **Results**: Fascicle length of the GM and VL was longer in post-PHV compared to pre-PHV athletes (*d* = 1.50 and *d* = 2.22, respectively, *p* < 0.001). Differences between circa and post-PHV athletes were observed only in GM (*d* = 0.84, *p* = 0.005). TH showed progressively greater values in both muscles across maturity stages (*p* < 0.001). PA was larger in post- compared to pre-PHV athletes, only in GM (*p* = 0.009). When all athletes were examined as one group, correlations were found between anthropometrics, maturity index and muscle architecture (*r* = 0.164–0.744 and 0.284–0.622, respectively, *p* < 0.05). In addition, in the GM, body mass and training experience accounted for 40% of the variance in FL (*p* < 0.001), whereas body mass and FL explained 66% of the variance in TH (*p* < 0.001). In the VL, stature and body mass explained 49% of the variance in FL (*p* < 0.001), while body mass, fascicle length, and maturity index explained 68% of the variance in TH, with maturity exhibiting a negative coefficient (*p* < 0.001). **Conclusions**: FL and TH of both muscles demonstrated larger values between pre- and post-PHV. GM and VL exhibited different morphological patterns, probably due to bone development and loading. Athletes’ body mass best predicted FL in both muscles, whereas the greater values in TH in post- compared to pre-PHV athletes appear to be associated with body mass and FL. The influence of maturity on VL thickness diminishes at more advanced stages of development.

## 1. Introduction

Skeletal muscle architecture is defined as the arrangement of muscle fibers within a muscle relative to the axis of force generation and is a primer determinant of muscle function [1]. Key architectural characteristics are fascicle length and angle and muscle thickness, all of which affect force–length and force–velocity relationships [2,3,4]. For example, increases in pennation angle and muscle thickness are generally associated with greater force-producing capacity via increases in physiological cross-sectional area, whereas longer fascicles relate more directly to higher contraction velocity and potential power output [5,6].

Growth and mechanical loading (e.g., exercise training) induce increases in muscle volume and morphological adaptations [7,8,9]. Comparative studies examining muscle architecture in children and adolescents are limited, particularly in relation to both chronological age and biological maturity. During maturation, the neuromuscular system develops in a non-linear manner, resulting in considerable individual variability in muscle characteristics and performance [10,11]. As yet, most available evidence on muscle architecture comes from cross-sectional studies with small numbers of participants that reported comprehensive data for children and adolescents aged 9–19 or 5–12 years, irrespective of their maturity stage [12,13]. However, it is well established that children of the same chronological age may differ markedly in maturity. Consequently, how muscle architecture differs across maturity stages remains largely unknown. One previous longitudinal study examined muscle architecture of the lower limbs between pre-, circa- and post-peak height velocity boys, and reported large increases from pre- to post-peak height velocity in gastrocnemius medialis and vastus lateralis fascicle length and angle and muscle thickness [14]. In another study, Panidi et al. reported that older age groups of female volleyball players (8–14 years) had greater fascicle length and anatomical cross-sectional area compared with younger age groups [15]. However, as this was a cross-sectional study, the observed differences reflect comparisons between age groups rather than longitudinal changes within individuals [15]. Similarly, Kubo et al. [8] described differences across ages in boys but did not establish direct growth trajectories. Nevertheless, evidence on muscle morphology in female athletes across different maturity stages remains limited, although different maturity stages may reflect underlying factors—such as hormonal fluctuations, body composition, neuromuscular development, and skeletal maturation—that contribute to the differences observed between groups.

Volleyball is a weight-bearing sport involving repeated jumping and rapid directional changes [16,17]. Volleyball training places substantial demands on lower-limb muscles such as the gastrocnemius and vastus lateralis, which may differ in loading patterns. Furthermore, different muscles produce varying amounts of force during jumping and may therefore exhibit variability in their architectural characteristics [18]. Despite the importance of muscle architecture for functional performance, little is known about how muscle morphology differs between maturity stages—particularly in female athletes, who are often understudied. This gap is further widened by the frequent lack of examination of children approaching peak height velocity, a critical phase of rapid growth. Thus, examining differences in muscle morphology between maturity groups may provide insight into patterns of muscle morphology associated with maturation and inform age- and maturity-appropriate training strategies [15,19]. Therefore, the aim of this cross-sectional study was to compare gastrocnemius medialis and vastus lateralis muscle architecture in female athletes at varying maturity stages.

## 2. Materials and Methods

### 2.1. Participants

One hundred and forty-four female volleyball players aged 8–18 years were recruited for this study. The athletes had between 1 and 7 years of training experience and were assigned to one of the following three maturity groups: pre-peak height velocity (PHV), circa-PHV, and post-PHV. All volleyball athletes were from the same club, under a single coach, following a structured program applied consistently to pre-, circa- and post-PHV athletes. Athletes followed volleyball training according to their age group four times per week (60–90 min in each training session). In youth female volleyball, typical training primarily targeted technical and tactical skills, with minimal strength or conditioning at early stages. Older age groups of players engaged in higher training intensity and physical demands, including structured strength or conditioning exercises 1–2 times per week.

Exclusion criteria were training experience of less than one year and any lower limb injury over the past 6 months. Parental informed consent, subjects’ oral, and written consent were collected for children and adolescent athletes. The study design and procedures followed the declaration of Helsinki. The Institutional Ethics Committee approved the study (registration number: 1644/15-05-2024).

### 2.2. Experimental Design

To examine differences between pre-, circa- and post- PHV athletes, in gastrocnemius medialis (GM) and vastus lateralis (VL) muscle architecture (fascicle length: FL; pennation angle: PA; muscle thickness: TH), in 144 female volleyball athletes were assessed, via ultrasonography. Athletes visited the laboratory twice. On the first visit, athletes’ anthropometric characteristics were assessed, and on the second, GΜ and VL muscle architecture was assessed. All assessments were conducted in the morning, at a room temperature of 21–23 °C. Before ultrasonography, athletes lay on a physiotherapy bed for 20 min to allow fluid redistribution. In addition, all athletes were instructed to avoid any exercise for the previous 48 h. Of the 144 athletes, 20 were assessed twice to calculate the intra-class correlation coefficient (ICC). The twenty athletes included for repeated measures were randomly selected from the total sample, ensuring representation across the three groups. The time interval between assessments was two days.

### 2.3. Anthropometric Characteristics

Anthropometric parameters were measured to assess maturation and growth. A stadiometer was used to measure stature (cm) (Seca 208, Hamburg, Germany). Sitting height (cm) was measured using a hand-made scale on the wall, and body mass (kg) was measured with a calibrated digital scale (Seca 710, Hamburg, Germany). Femur length (cm) was measured as the distance between the trochanter major and the tibiofemoral joint. Calf length (cm) was measured as the distance between the tibiofemoral joint and the medial malleolus’s most prominent point. Maturity offset was calculated using the modified maturity offset equation for girls [20], estimating the years from PHV based on stature and chronological age. Subsequently, athletes were categorized into pre-PHV (≥1 year before PHV), circa-PHV (±1 year around PHV), and post-PHV (≥1 year after PHV) [20,21].

The body mass index was calculated as the ratio of body mass to the square of stature (kg/m^2^). The characteristics of the participants are shown in Table 1.

### 2.4. Ultrasonography

Extended field-of-view (EFOV) imaging using a 10 MHz linear probe (38 mm) was used to assess muscle architecture of the gastrocnemius medialis and vastus lateralis at rest (product model Z5, Shenzhen, Mindray Bio-Medical Electronics Co., Ltd., Shenzhen, China). The EFOV technique avoids trigonometric estimations utilizing non-linear aponeuroses by capturing fascicles longer than the probe’s length.

**Figure 1:**
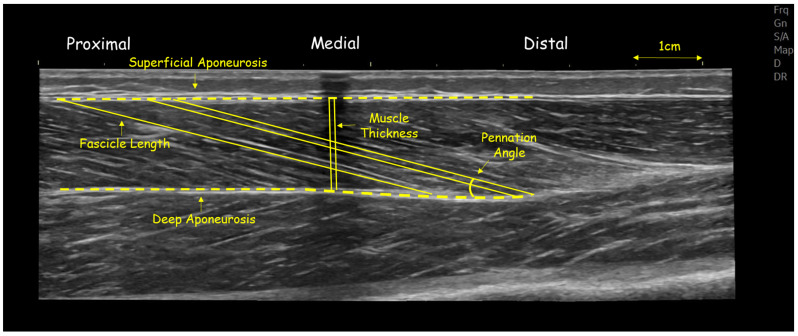
Panoramic ultrasound image of gastrocnemius medialis of a volleyball athlete, showing fascicle length and angle and muscle thickness at mid-belly.

The gastrocnemius medialis and vastus lateralis were chosen for their functional relevance in volleyball—contributing to jumping, sprinting and change of direction performance—and their sensitivity to maturity-related adaptations. All scans were performed by the same experienced operator, with image analysis conducted blinded to participant identity and maturity group. First, athletes lay prone on the physiotherapy bed with their ankle joints off the edge to measure gastrocnemius medialis architecture [15]. FL, PA, and TH were measured at the midpoint of gastrocnemius medialis (Figure 1). The midpoint of the gastrocnemius medialis muscle belly, corresponding to approximately 30% of the distance between the tibiofemoral joint and the most conspicuous location of the medial malleolus, was marked with an echo-absorptive marker [15]. To obtain a panoramic image parallel to the orientation of the fascicles, a permanent pen was used to create a probe path on the skin using real-time imaging [17]. Acoustic penetration was provided using aquasonic gel, and the probe moved slowly and steadily along the muscle while imposing steady low pressure [15].

**Figure 2:**
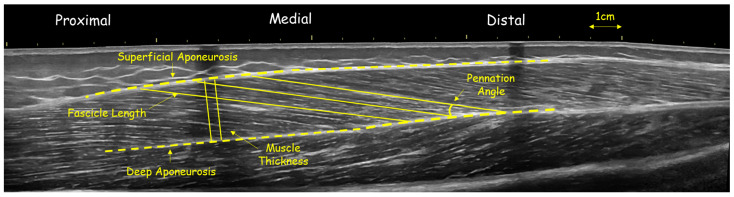
Panoramic ultrasound image of vastus lateralis of a volleyball athlete, showing fascicle length and angle and muscle thickness at mid-belly.

Similarly, to assess the muscle architecture of vastus lateralis, athletes laid in a supine position, with the hip angle at 180°, while the knee angle was at approximately 170°. The distance from the greater trochanter to the external condyle was first measured. The midpoint (50% of this distance) was then marked, following the same procedure used for the gastrocnemius muscle. A tape was placed at this midpoint to indicate the central region of the muscle. The area of interest was outlined and modeled by tracing waypoints along the length of the head, both before and after the 50% marker. Panoramic imaging was also used for this muscle with continuous and steady probe movement along the prescribed path to obtain a single composite image (Figure 2). In addition, the slope was adjusted according to each architectural structure to minimize errors in the projections of the bundles and/or their parallax [22]. In each capture, three distinct fascicles crossing the marker at the center of the muscle were measured, and their average value was used for further analysis (Figure 1 and Figure 2). The outline of the muscle area was measured manually, with image analysis software using a polygon selection tool (Figure 2) (Motic Images Plus 2.0, Motic, Hong Kong, China). Similar approaches are widely used to assess gastrocnemius medialis and vastus lateralis architecture in growing populations [4,9,12,22]. Test–retest reliability using ICC (two-way ANOVA random effects model) for muscle FL was (0.971; 95% CI: 0.902–0.991), PA was (0.921; 95% CI: 0.898–0.929) and TH was (0.992; 95% CI: 0.979–0.995).

### 2.5. Statistical Procedures

SPSS statistical software (IBM SPSS Statistics Version 29.0, IBM Corporation, Armonk, NY, USA) was used to conduct statistical analyses. For all examined variables, descriptives were calculated. The Kolmogorov–Smirnov test was used as a preliminary check of raw data normality. To investigate age group differences, a one-way ANOVA (group) was performed independently for anthropometrics and muscle architecture. Tukey’s post hoc test was used when a significant main effect was found (*p* < 0.05). Partial eta squared (η^2^) was used to calculate the effect sizes (ES) for the ANOVA (small: 0.01 to 0.059; moderate: 0.06 to 0.137; large: >0.138). Normality for ANOVA was checked using Shapiro–Wilk tests and visual inspection of Q–Q plots of the residuals, while homogeneity of variances was verified with Levene’s test. These diagnostics confirmed that the assumptions for ANOVA were adequately met. For pairwise comparisons, effect sizes were calculated using Cohen’s *d* statistical index (trivial: 0–0.19; small: 0.20–0.49; moderate: 0.50–0.79; large: 0.80 and greater) [23]. To assess test–retest reliability, the intra-class correlation coefficients (ICCs) were used. Pearson’s correlation coefficient (*r*) [24] examined linear associations between the examined variables and was regarded as trivial (*r* < 0.10), small (*r* = 0.10–0.29), moderate (*r* = 0.30–0.49), large (*r* = 0.50–0.69), very large (*r* = 0.70–0.89) or almost perfect (*r* ≥ 0.90) [25]. Stepwise multiple regression analyses (backward elimination) were used to examine which variables (anthropometrics, maturity index and in addition for TH, fascicle length) contributed most significantly to gastrocnemius medialis and vastus lateralis FL and TH. Model stability was monitored, and only predictors that consistently contributed to the model were retained. For regression analyses, assumptions were also checked: residuals were normally distributed (Shapiro–Wilk test and Q–Q plots), linearity between predictors and outcomes and homoscedasticity were confirmed from residual-versus-fitted plots, and multicollinearity was assessed using Variance Inflation Factor (VIF), with all values <5. These diagnostics indicated that the assumptions for both ANOVA and regression analyses were adequately met.

## 3. Results

### 3.1. Muscle Architecture

**Figure 3:**
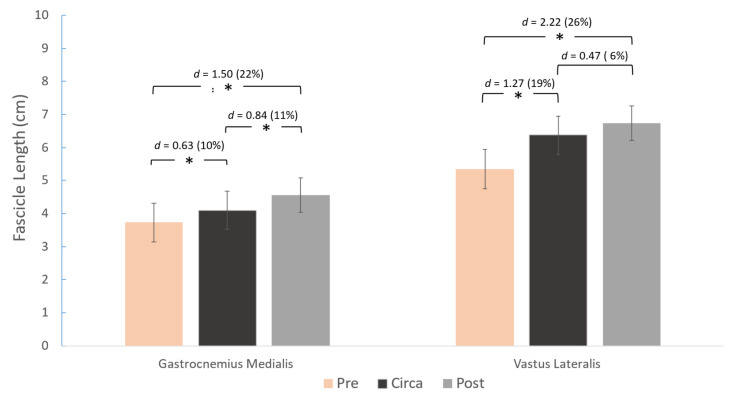
Differences between the three maturity groups for gastrocnemius medialis (left) and vastus lateralis (right) fascicle length and pairwise comparisons (Cohen’s *d*). * *p* < 0.05.

**Figure 4:**
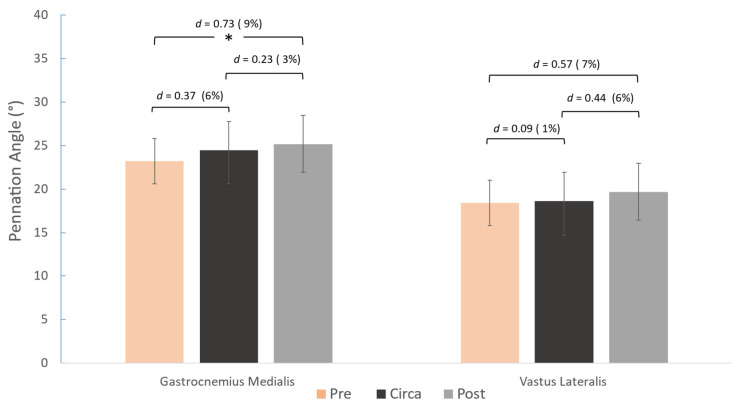
Differences between the three maturity groups for gastrocnemius medialis (left) and vastus lateralis (right) pennation angle and pairwise comparisons (Cohen’s *d*). * *p* < 0.05.

**Figure 5:**
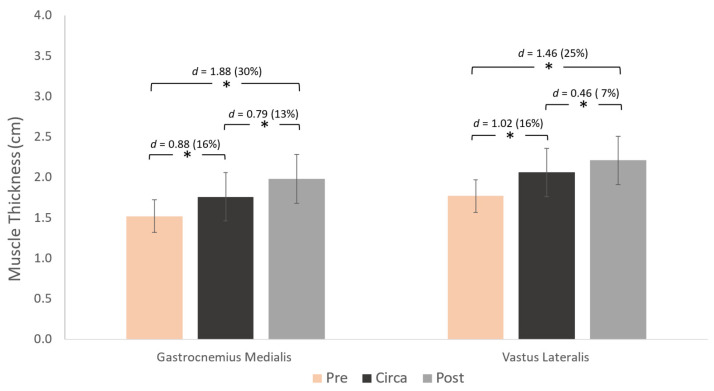
Differences between the three maturity groups for gastrocnemius medialis (left) and vastus lateralis (right) muscle thickness and pairwise comparisons (Cohen’s *d*). * *p* < 0.05.

A main effect for group was found for gastrocnemius medialis FL (*p* < 0.001), PA (*p* = 0.009), and TH (*p* < 0.001) (Figure 3, Figure 4 and Figure 5). Similarly, a main effect for group was found for vastus lateralis FL (*p* < 0.001) and TH (*p* < 0.001), but not for PA (*p* = 0.069) (Figure 3, Figure 4 and Figure 5). Post-hoc comparisons showed that gastrocnemius FL and vastus lateralis FL were greater in pre- compared to circa-PHV athletes (*p* = 0.005 and *p* < 0.001, respectively). FL was also longer in pre- to post-PHV athletes (*p* = 0.001 and *p* = 0.001, respectively) for both muscles. However, FL was longer in circa-PHV compared to post-PHV athletes only in the gastrocnemius medialis (*p* < 0.001), but not in the vastus lateralis (*p* = 0.114) (Figure 3).

For PA, post-hoc comparisons revealed that PA was greater only in the GM from pre- to post-PHV (*p* = 0.009), with no differences observed across the other maturity stages. No differences were observed in the PA of VL.

Gastrocnemius medialis and vastus lateralis TH was greater from pre- to circa-PHV (*p* < 0.001 and *p* < 0.001, respectively). It was also greater in pre- compared to post-PHV athletes (*p* = 0.001 and *p* = 0.001, respectively) for both muscles.

### 3.2. Correlations

**Figure 6:**
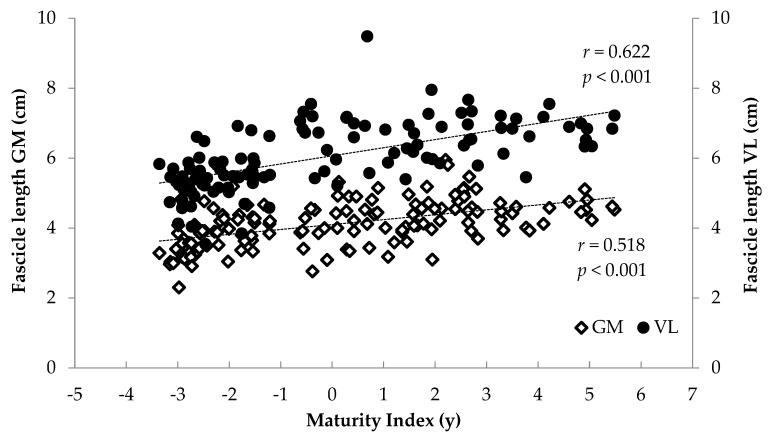
Association between athletes’ maturity index and gastrocnemius medialis (GM) and vastus lateralis (VL) fascicle length.

**Figure 7:**
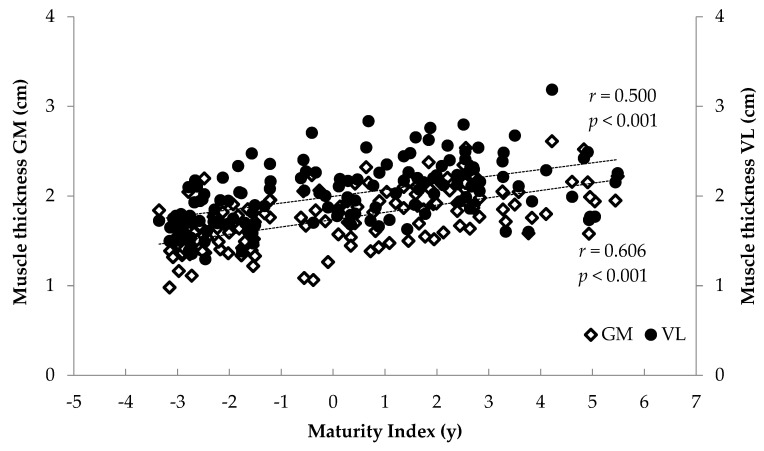
Association between athletes’ maturity index and gastrocnemius medialis (GM) and vastus lateralis (VL) muscle thickness.

**Figure 8:**
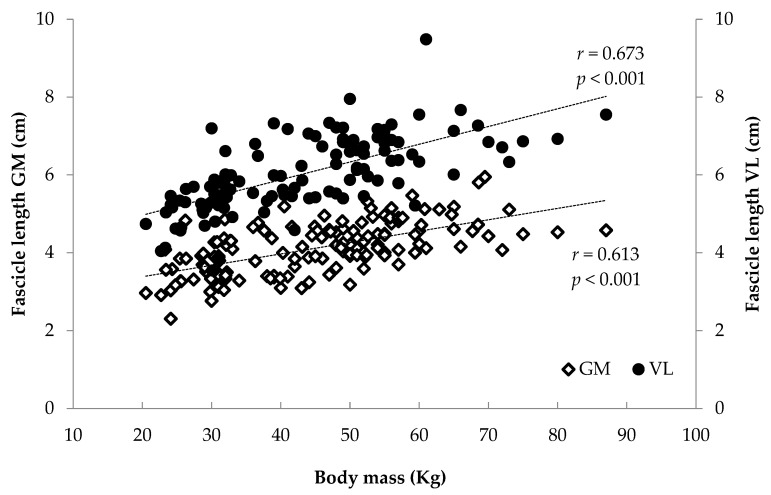
Association between athletes’ body mass and gastrocnemius medialis (GM) and vastus lateralis (VL) fascicle length.

**Figure 9:**
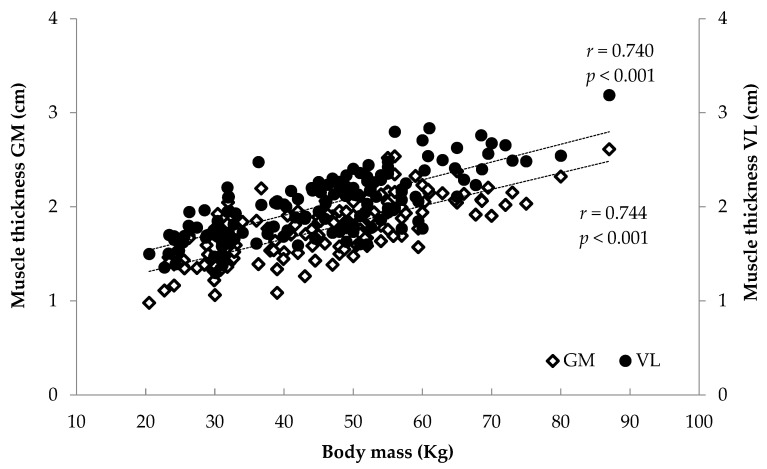
Association between athletes’ body mass and gastrocnemius medialis (GM) and vastus lateralis (VL) muscle thickness.

When all athletes were examined together, significant associations were found between anthropometrics (body mass, stature, BMI, femur length, calf length and sitting height) and FL and TH (*r* = 0.375 to 0.744, *p* < 0.001) and between age and maturity index and muscle architecture (*r* = 0.284 to 0.622, *p* < 0.001) (Figure 6, Figure 7, Figure 8 and Figure 9). Additionally, significant associations were found between gastrocnemius medialis PA and anthropometrics (stature, body mass, sitting height, calf length) (*r* = 0.202 to 0.424, *p* < 0.01) and with age and maturity index (*r* = 0.296 and 0.284, respectively, *p* < 0.001). PA of vastus lateralis was associated only with body mass, stature, sitting height and BMI (*r* = 0.204 to 0.319, *p* < 0.01).

### 3.3. Regression Analyses

Stepwise multiple regression analyses examined which variables contributed most significantly to gastrocnemius medialis and vastus lateralis FL. For muscle thickness, in addition to anthropometrics and maturity index, FL was included in the regression, as muscle thickness may also be influenced by changes in FL.

Body mass and training experience explained 40% of the variance in GM fascicle length (AR^2^ = 0.40, F = 48.590, *p* < 0.001) with a positive coefficient (β = 0.027 and β = 0.076, *p* < 0.007).

Body mass and stature were included in the multiple regression analysis for vastus lateralis FL, explaining the 49% of the variance (AR^2^ = 0.49, F = 57.144, *p* < 0.001) with a positive coefficient (β = 0.021 and β = 0.027, *p* < 0.014). Assumption checks confirmed model adequacy: collinearity diagnostics showed VIF values of 2.747 for both predictors, well below the threshold of concern (all VIF < 3). These results indicate that higher body mass and stature are independently associated with longer fascicle length of the vastus lateralis.

Furthermore, body mass, FL and maturity index were included in the multiple regression analysis for gastrocnemius medialis TH explaining the 68% of the variance (AR^2^ = 0.68, F = 82.958, *p* < 0.001). Body mass had a significant positive association (β = 0.018, *p* < 0.001), as well as FL (β = 0.174, *p* < 0.001), while maturity index showed a negative association (β = −0.050, *p* < 0.001). Model diagnostics supported adequacy: all VIF values were <5, showing no evidence of multicollinearity.

## 4. Discussion

The aim of this cross-sectional study was to examine differences in gastrocnemius medialis and vastus lateralis muscle architecture in pre-, circa- and post-PHV female volleyball athletes. GM and VL fascicle length were longer in post-PHV compared to pre-PHV athletes, while differences between circa and post-PHV athletes were observed only in GM. In both muscles, TH was greater across each maturation stage. PA was larger in post- compared to pre-PHV athletes, only in GM. Significant correlations were found between anthropometric characteristics, maturity index and muscle architecture. Athletes’ body mass predicted FL in both muscles, whereas differences in TH across maturity stages appear to be associated with body mass and FL. The influence of maturity on vastus lateralis TH diminishes at more advanced stages of development.

The period around PHV age is a crucial phase for muscle development, characterized by rapid somatic development and elevated anabolic hormones that enhance protein synthesis and facilitate substantial gains in muscle mass and strength [26]. The results of this study indicated that FL of GM and VL were 22 and 26% longer, respectively, in the post-PHV compared to the pre-PHV group. Radnor et al. [14], in a longitudinal study, also reported that post-PHV males had longer VL fascicles compared to pre-PHV males (*d* = 0.66). However, no significant differences in gastrocnemius medialis FL were reported between maturity groups in another cross-sectional study [27]. A possible explanation for the differences between studies may be the variation in age range. Radnor’s study [27] included secondary school children (typically aged 11–18 years), whereas this study covered a broader age range of 8–18 years, thus possibly better representing the full spectrum of development. Furthermore, there is the possibility that volleyball training affected fascicle length in the participants of this study, given the repetitive take-off and landing skills, involving eccentric contractions and substantial dorsiflexion inherent to the sport [16]. In another study that examined female volleyball athletes according to their chronological age, gastrocnemius medialis FL was longer at the age of 14 years compared to the age of 8, while thereafter and until the age of 18 years, non-significant differences were observed [15]. Subtle differences were reported regarding FL in this study compared to that study, with significantly larger values in both muscles in post- compared to pre-PHV athletes (approximately 9–15.5 years of age, respectively), highlighting that biological maturity may better reflect the maturity-related differences in muscle architecture. Collectively, it seems that the fascicles reach adult length by the age of 15, after which only minimal differences are observed. Kubo et al. [4] found no differences in GM architecture between adults and 15-year-old adolescent males, and the authors assumed that muscle architecture reaches adult-like characteristics by mid-adolescence. In line with this, a study on adult female volleyball players reported gastrocnemius medialis FL [28] comparable to that of the post-PHV athletes of the present study (4.74 ± 0.33 cm and 4.56 ± 0.52 cm, respectively).

Notably, from pre- to circa-PHV, a large difference was observed in VL fascicle length (19%) compared to GM (10%), accompanied by a non-significant difference in VL, and a similar difference in GM (11%) from circa- to post-PHV, thus indicating that the fascicles of the two muscles exhibit different patterns of adaptation across maturation stages. The twofold greater difference in vastus lateralis FL compared to GM up to circa-PHV suggests that the FL of VL exhibits greater absolute variation between pre-PHV and circa-PHV. This likely reflects an adaptation to accommodate the rapid longitudinal growth of the femur during this critical developmental period, implying that muscle length adjusts to bone growth to preserve the force–length relationship. The gastrocnemius medialis displays a consistent pattern of fascicular length after the time of peak growth, which may be attributed to anatomical configuration and mechanical loading characteristics. It should be noted that this pattern may be unique to volleyball athletes due to their somatotypes with longer femurs and high jumping demands.

Muscle thickness differences were observed between maturation stages in both muscles. Large differences of 30 and 25% were observed from pre- to post-PHV for GM and VL, respectively, and by 13 and 7% from circa- to post-PHV. Radnor et al. [14] also reported large differences in gastrocnemius medialis and vastus lateralis TH between pre- and post-PHV boys, showing that the pre- to post-PHV period is critical for TH development, associated with rapid growth and hormonal changes that support tissue adaptation and strength gains.

Although the study of Radnor did not report differences in gastrocnemius medialis TH between circa- and post-PHV boys, we observed significant differences during this time frame. Albeit speculatively, these differences may be attributed to the GM’s anatomical attachment, greater pennation angle, and the specific mechanical loading of the sport, variables which were not measured in this study. Binzoni et al. [29] examined age-related differences in gastrocnemius medialis muscle architecture, observing variations in architectural parameters such as pennation angle across ages. Subsequent empirical work in children aged 5–12 years also demonstrated age-associated differences in muscle thickness, estimating an approximate 7% difference by year [9]. Furthermore, the positive correlations between the thicknesses of GM and VL (r = 0.661, *p* < 0.001) support the notion that these muscles tend to vary in a related manner during maturation.

Pennation angle values were larger by 9% between pre-PHV and post-PHV athletes only in the GM, with no respective difference observed in the VL. In contrast, Radnor et al. found significant differences in PA of both muscles between pre-PHV and post-PHV boys; however, the difference in vastus lateralis PA fell within the typical error and was therefore considered non-significant. Another study also reported that the PA of VL did not differ significantly between boys aged 11 years and 14 years or into adulthood [8]. This result is interesting because in adults, both TH and PA are linked to force and power production [30,31,32]. The differences in TH and FL were observed alongside relatively stable PA values, which may suggest a developmental pattern distinct from adults, but without inferring mechanistic optimization or causal relationships.

Significant correlations were found between FL, PA and TH with maturity index and anthropometrics—in particular body mass, and stature—highlighting the link between muscle growth and body dimensions due to maturation [7,9,15]. Skeletal muscle geometry is closely associated with body dimensions that impose mechanical loading, including stretch and load, which result from differences in limb length and body weight, respectively. Furthermore, significant correlations were found between age and maturity index and muscle architecture. Taken together, these associations suggest that growth-related factors are associated with variation in muscle architectural characteristics. Growth is a dominant stimulus for musculoskeletal adaptation, exerting a profound influence and guiding the architectural and functional maturation of the neuromuscular system [29]. Based on these associations, regression analyses were conducted. In the medial gastrocnemius, 40% of FL variance was explained by body mass and training experience, while 67% of TH variance was explained by body mass and FL. In the vastus lateralis, 49% of FL was explained by stature and body mass, while 68% of TH was explained by body mass, FL and maturity index. The finding that body mass predicts FL in both muscles is in line with previous studies reporting relationships between body size and muscle architectural properties [29,33]. Although cross-sectional, these studies suggest that greater body mass may be linked to differences in muscle fascicle length and thickness due to greater mechanical loading and metabolic demands [29,33]. Body mass is not only an index of muscle tissue quantity but also acts as an endogenous mechanical load that may promote structural adaptations such as increased FL, PA and TH. For GM, in addition to body mass, training experience also predicted FL, possibly reflecting long-term morphological adaptations, especially in sports like volleyball that involve repeated eccentric contractions through a wide range of motion in the lower limbs [15,16,17,34]. Furthermore, both stature and body mass predicted fascicle length of VL, suggesting coordinated growth patterns of both muscular and skeletal tissue [12].

Regarding TH, aside from body mass, FL was linked to the overall volume of the gastrocnemius and VL, implying that fascicles may contribute to differences in muscle thickness, in addition to general hypertrophic effects. Since differences in muscle thickness across maturity stages were found in this study without further differences in FL or PA, this may indicate a potential contribution of non-contractile tissues (e.g., fasciae, epimysium, perimysium) [35].

The negative association between maturity index and FL of VL may reflect a plateau or weaker association at advanced stages. Malina et al. [36] further noted that hormonal factors during puberty drive structural adaptations, potentially at rates distinct from external influences. During childhood, growth is the primary driver of musculoskeletal adaptations, influencing neuromuscular development significantly more than training. As growth stabilizes, training load (i.e., volleyball or strength training) may become the dominant influence.

This study has some limitations that should be acknowledged. First, the cross-sectional study design precludes determining within-subject changes over time and cannot establish causal relationships between interventions or aging and muscle morphology. Longitudinal data or studies using robust imaging techniques (i.e., magnetic resonance imaging) could better identify periods of accelerated growth and changes in muscle morphology, offering insight into the timing and tempo of the pubertal growth spurt [36,37,38,39]. This limitation should be considered when interpreting maturity-related findings [38]. In addition, although all athletes trained at the same club under a single coach, differences in the training content across maturity stages likely influence muscle architecture and should be considered when interpreting group differences. Cohen’s *d* with pooled standard deviations was used as a straightforward and widely reported effect size for post-hoc contrasts and pairwise comparisons. It should be noted that model-based estimates using marginal means and standard errors can be more robust, especially with unequal group sizes or heteroscedasticity. Lastly, although PHV estimated from body dimensions provides a useful proxy, it is not a definitive index of biological maturity, as it may not fully capture individual variability.

Nevertheless, to date, no large-scale study has examined muscle architecture in developing female athletes, despite their growing participation in sport. A key strength of this cross-sectional study is the inclusion of 144 female youth athletes, which provides sufficient statistical power to characterize variability in muscle morphology and generate hypotheses for future longitudinal research. The findings suggest that the circa-PHV stage is a critical time frame for musculoskeletal development, highlighting potential implications for individualized training strategies to optimize performance and reduce injury risk.

## 5. Conclusions

Significant differences in fascicle length and muscle thickness were observed across maturity stages, with values varying across stages around peak height velocity. The gastrocnemius medialis and vastus lateralis differed in their patterns of fascicle length and thickness across these groups, likely reflecting interactions between bone development and loading patterns. Athletes’ body mass was the strongest predictor of FL in both muscles, whereas TH was associated with both body mass and FL. The association between maturity and VL thickness was weaker at more advanced maturity stages.

## Figures and Tables

**Table 1:** Anthropometric characteristics of the participants (means ± standard deviations).

	Pre-PHV (*n* = 50)	Circa-PHV (*n* = 45)	Post-PHV (*n* = 49)	*p*
Age (y)	9.2 ± 0.6	11.9 ± 1.0	15.3 ± 1.5	<0.001
Stature (cm)	134.8 ± 6.2	154.9 ± 6.9	165.5 ± 6.1	<0.001
Sitting height (cm)	71.7 ± 3.5	80.6 ± 5.1	88.6 ± 3.5	<0.001
Leg length (cm)	69.1 ± 4.2	78.3 ± 5.1	84.2 ± 5.9	<0.001
Femur length (cm)	32.6 ± 3.2	41.0 ± 4.6	43.0 ± 4.8	<0.001
Calf length (cm)	30.8 ± 2.4	36.5 ± 2.8	39.2 ± 2.1	<0.001
Weight (kg)	31.1 ± 5.9	48.1 ± 9.1	57.4 ± 9.4	<0.001
BMI (kg/m^2^)	17.0 ± 2.1	20.0 ± 3.2	21.0 ± 3.4	<0.001
PHV (y)	11.7 ± 0.3	11.8 ± 0.3	12.3 ± 0.6	=0.099
Maturity offset (y)	−2.5 ± 0.5	0.1 ± 0.9	3.0 ± 1.1	<0.001

## Data Availability

Data are available from the first author upon reasonable request due to ethical reasons.

## Abbreviations

The following abbreviations are used in this manuscript:

PHV	Peak Height Velocity
FL	Fascicle Length
PA	Pennation Angle
TH	Muscle Thickness
GM	Gastrocnemius Medialis
VL	Vastus Lateralis
